# Evaluation of the Diagnostic Sensitivity of the VIASURE *Leishmania* Real-Time PCR Detection Kit Prototype for the Diagnosis of Cutaneous and Visceral Leishmaniasis

**DOI:** 10.1155/2023/1172087

**Published:** 2023-12-18

**Authors:** Albert Arnau, Alba Abras, Cristina Ballart, Anna Fernández-Arévalo, Mary Cruz Torrico, Silvia Tebar, Teresa Llovet, Montserrat Gállego, Carmen Muñoz

**Affiliations:** ^1^Secció de Parasitologia, Departament de Biologia, Sanitat i Medi Ambient, Facultat de Farmàcia i Ciències de l'Alimentació, Universitat de Barcelona, Barcelona, Spain; ^2^Àrea de Genètica, Departament de Biologia, Universitat de Girona, Girona, Spain; ^3^Institut de Salut Global de Barcelona (ISGlobal), Hospital Clínic, Universitat de Barcelona, Barcelona, Spain; ^4^Facultad de Medicina, Universidad Mayor de San Simón (U.M.S.S.), Cochabamba, Bolivia; ^5^Servei de Microbiologia, Hospital de la Santa Creu i Sant Pau, Barcelona, Spain; ^6^Institut de Recerca Biomèdica Sant Pau, Barcelona, Spain; ^7^Departament de Genètica i Microbiologia, Universitat Autònoma de Barcelona, Barcelona, Spain; ^8^CIBERINFEC, ISCIII- CIBER de Enfermedades Infecciosas, Instituto de Salud Carlos III, Madrid, Spain

## Abstract

Leishmaniasis is a parasitic disease with clinical presentations that vary from asymptomatic infection to cutaneous, mucocutaneous, or visceral disease. Global change, with migratory movements and travels, among others, has had an impact on the presentation of the clinical forms of leishmaniasis in a given area, hindering its diagnosis. The traditional parasitological techniques have limited sensitivity, and currently, there is no reference or gold-standard molecular diagnostic method. The aim of this study was to evaluate the effectivity of the VIASURE *Leishmania* Real-Time PCR Detection Kit prototype (CerTest Biotec, Zaragoza, Spain) for the diagnosis of autochthonous and imported leishmaniasis in comparison with two other commercialized molecular kits: STAT-NAT® *Leishmania* spp. (Sentinel, Milano, Italy) and *Leishmania* spp. Real-TM PCR Kit (Sacace Biotechnologies, Como, Italy). Four species of *Leishmania*, *L. infantum*, *L. major*, *L. braziliensis*, and *L. panamensis*, were targeted to assess analytical sensitivity, whereas diagnostic sensitivity was evaluated by studying a panel of 49 DNA samples from patients with suspected or confirmed *Leishmania* infection. The prototype could detect all the New and Old World species studied and achieved a limit of detection of 5 × 10^−5^ ng DNA/*μ*L in all species. Also, it allowed the diagnosis of autochthonous and imported cases of cutaneous and visceral leishmaniasis (VL). Diagnostic sensitivity was 81.8% for the prototype and 100% for the Sacace kit (27 and 33 positive samples detected, respectively). The STAT-NAT® kit failed to detect *Vianna* species. The VIASURE *Leishmania* Real-Time PCR Detection Kit prototype was found to have good analytical and diagnostic sensitivity. Using a simple protocol and ready-to-use reagents, results are obtained quickly and are easy to interpret. The evaluation results indicate that the test is a promising candidate for routine diagnosis of cutaneous leishmaniasis and VL in endemic countries, but more studies are necessary to address its sensitivity and specificity.

## 1. Introduction

Leishmaniasis is a vector-borne disease caused by protozoan parasites from the genus *Leishmania*, which are transmitted by the bite of infected female sandflies of the genera *Phlebotomus* and *Lutzomyia* [1, 2]. According to the World Health Organization, leishmaniasis is one of the seven most important tropical diseases worldwide. In 2020, it was described as endemic in large areas of the tropics, being present in around 98 countries and territories in all continents except Oceania [1].

About 20 species of *Leishmania* have been identified as pathogenic for humans [3], and they cause three main clinical variants of the disease: (i) cutaneous leishmaniasis (CL), with infected macrophages resident in the skin; (ii) mucosal leishmaniasis, which affects mucous membranes of the nose, mouth and throat; (iii) and visceral leishmaniasis (VL), with infected mononuclear phagocytic cells in the liver, spleen, bone marrow, lymphatics nodes, and intestine [3, 4].

The most common form is CL, with about 600,000 to 1 million new cases occurring annually worldwide [1]. CL is characterized by epithelial lesions and ulcers in exposed regions of the body [5]. The cutaneous, mucosal, and mucocutaneous forms of leishmaniasis, collectively referred to as tegumentary leishmaniasis (TL), are not usually fatal. The most severe form is VL, with 50,000 to 90,000 new cases reported per year [1]. Without treatment, VL is fatal in 95% of cases and, even when treated, is associated with case-fatality rates of 10%–20% [6, 7]. All the *Leishmania* species infecting humans can cause CL, whereas the species known to cause VL belong to the *L. donovani* complex [4, 8].

The diagnosis of leishmaniasis relies on clinical manifestations and epidemiological and laboratory data [9, 10]. In the absence of a gold-standard diagnostic test, different combinatorial algorithms are used. Methods to detect *Leishmania* infection include microscopy, *in vitro* culture, serology, dermal diagnostic tests, xenodiagnosis, and molecular approaches [11–14]. Each method has its advantages, and its usefulness may vary according to the clinical form of the disease [15–17].

Recently, several molecular tools have been developed for the diagnosis of leishmaniasis, and they are playing an increasingly relevant role in this field due to their high sensitivity, specificity, and applicability to a variety of clinical samples [13]. Molecular methods are also useful for monitoring treatment response in patients with leishmaniasis and other diseases [18, 19]. Among them, quantitative real-time PCR (qPCR) is increasingly the method of choice, as it is fast, has a broad dynamic range, and drastically reduces carryover contamination because there is no need to open reaction tubes for post-PCR analyses [13, 20, 21].

In some endemic areas, including countries of South America, leishmaniasis is caused by a wide variety of coexisting *Leishmania* species [3, 10, 22], whereas in others, such as Spain, only one autochthonous species is involved; nevertheless, with increased population movement associated with globalization, cases are being imported from South America, the Maghreb, and elsewhere [23]. The difficulty of designing an optimal method to detect all the circulating species hampers the diagnosis of the disease. Commercial molecular kits are available mainly for the diagnosis of VL, and very few allow the detection of all species capable of causing TL. CerTest Biotec (Zaragoza, Spain) has recently developed a prototype qPCR assay to detect and diagnose infection by *Leishmania* spp. in patients with signs of TL or VL. This prototype was designed to avoid time-consuming manipulation steps, thus reducing the risk of contamination, by incorporating lyophilized reagents for the qPCR assay.

In this context, the aim of this study was to perform a comparative analysis of three molecular diagnostic kits for leishmaniasis: the new VIASURE *Leishmania* Real-Time PCR Detection Kit prototype (CerTest Biotec, Zaragoza, Spain) and the commercialized STAT-NAT® *Leishmania* spp. (Sentinel, Milano, Italy) and *Leishmania* spp. Real-TM PCR Kit (Sacace biotechnologies, Como, Italy) kits.

## 2. Materials and Methods

### 2.1. *Leishmania* Strains

To evaluate the analytical sensitivity of the diagnostic methods, four strains of four species of *Leishmania* were targeted. They belonged to the subgenus *Leishmania* from the Old World (*L. infantum* and *L. major*) and subgenus *Viannia* from the New World (*L. braziliensis* and *L. panamensis*). The strains were isolated from patients with CL or VL and cryopreserved in the Cryobanc of Trypanosomatids (Universitat de Barcelona). Before the analysis, the strains were thawed and cultured on Schneider's insect medium supplemented with 20% fetal bovine serum and 1% sterile human urine [23].

The DNA extraction from cryopreserved strains was carried out with a manual extraction kit for tissue samples (High Pure PCR Purification Kit of Roche, Basel, Switzerland) following the manufacturer's instructions. The extracted DNA was stored properly at −20°C until use. The DNA of the strains was quantified with a NanoDrop 1,000 Spectrophotometer (Waltham, Massachusetts, United States). DNA concentration was adjusted to around 50 ng/*µ*L of *Leishmania* DNA for each species, and 1/10 serial dilutions up to 5 × 10E−5 ng/*µ*L were carried out in the elution buffer supplied with the DNA extraction kit (Roche). The *Leishmania* strains were characterized by *hsp70* gene sequencing and MALDI-TOF MS [23].

### 2.2. DNA Samples

For the diagnostic sensitivity study, a panel of 49 DNAs was used, previously isolated from clinical samples of patients with suspected or culture-confirmed leishmaniasis without any clinical or epidemiological criteria. The panel of clinical samples consisted of 44 DNAs from skin biopsy samples and five DNAs from visceral clinical samples (one from peripheral blood, one from liver biopsy, and three from bone marrow aspiration). The DNA of clinical samples was obtained retrospectively using two different methods: the aforementioned manual extraction kit (Roche) and an advanced automated instrument with the EZ1 DNA Tissue or EZ1 DNA Blood Kits (Qiagen, Hilden, Germany). The extracts were stored at −20°C until analysis. The *Leishmania* isolates were obtained and characterized prior to the study.

### 2.3. Molecular Assays

The performance of the new prototype (VIASURE *Leishmania* Real Time PCR Detection Kit, CerTest) was compared with that of two commercial CE-marked kits already available for the molecular diagnosis of leishmaniasis: (i) the STAT-NAT® *Leishmania* spp. Kit (Sentinel Diagnostics), and (ii) the *Leishmania* spp. Real-TM PCR Kit (Sacace Biotechnologies). All the qPCR reactions were carried out in a QuantStudio 6 qPCR Flex system (Applied Biosystems, Waltham, Massachusetts, United States). All samples were tested in duplicate, including the positive and negative controls supplied by the respective kit, in each run.

The VIASURE kit is a prototype multiplex qPCR method designed for the detection of *Leishmania* spp. in different biological samples from patients with signs and symptoms of leishmaniasis using the 18S rRNA gene as a target. The amplification conditions were: one cycle at 2′ at 95°C, 45 cycles of 10′ at 95°C and 50′ at 60°C. The kit can be stored at room temperature. The results were analyzed using the software of the real-time PCR equipment according to the manufacturer's instructions. A sample was considered positive when the cycle threshold (Ct) value obtained was less than 40 (web page of the Certest prototype for more information https://www.certest.es/wp-content/uploads/2021/07/VIASURE_LEI_ES.pdf).

The STAT-NAT® (Sentinel) kit is a qualitative multiplex test based on qPCR amplification using fluorescent probes specific for *Leishmania* spp. According to the manufacturer's manual, the assay is able to detect all *Leishmania* species in whole blood samples. The kit allows room-temperature transport and storage. A sample was considered positive when the Ct value obtained was less than 40.

The Sacace kit is a qualitative diagnostic test, which, according to the manufacturer's manual, is able to detect all *Leishmania* species in tissue specimens such as skin sores (for CL) or bone marrow (for VL). The kit needs to be stored at −20°C. The extraction control could only be added in the assays with cryopreserved strains and not in the clinical analysis, which was carried out directly with stored DNAs. A sample was considered positive when the Ct value obtained was less than 40.

### 2.4. Data Analysis

The criteria for interpreting the test results were the following: samples were classified as positive when a confirmatory positive result was obtained by culture and/or by the commercial Sacace kit and were considered negative when a negative result was obtained with both techniques.

The following measures of diagnostic accuracy were calculated (TP, true positive; TN, true negative; FP, false positive; FN, false negative): sensitivity (calculated as TP/(TP + FN) × 100); specificity (calculated as TN/(TN + FP) × 100), positive and negative predictive values (PPV and NPV, respectively), which are the proportion of correctly diagnosed individuals with positive (PPV) or negative (NPV) results (calculated as TP/(TP + FP) × 100 and TN/(TN + FN) × 100, respectively), and the prevalence (calculated as (total positive/total samples) × 100). Calculations were performed with the software VassarStats, which is available online at http://vassarstats.net.

## 3. Results

### 3.1. Linearity of the Assays and Limit of Detection

**Figure 1:**
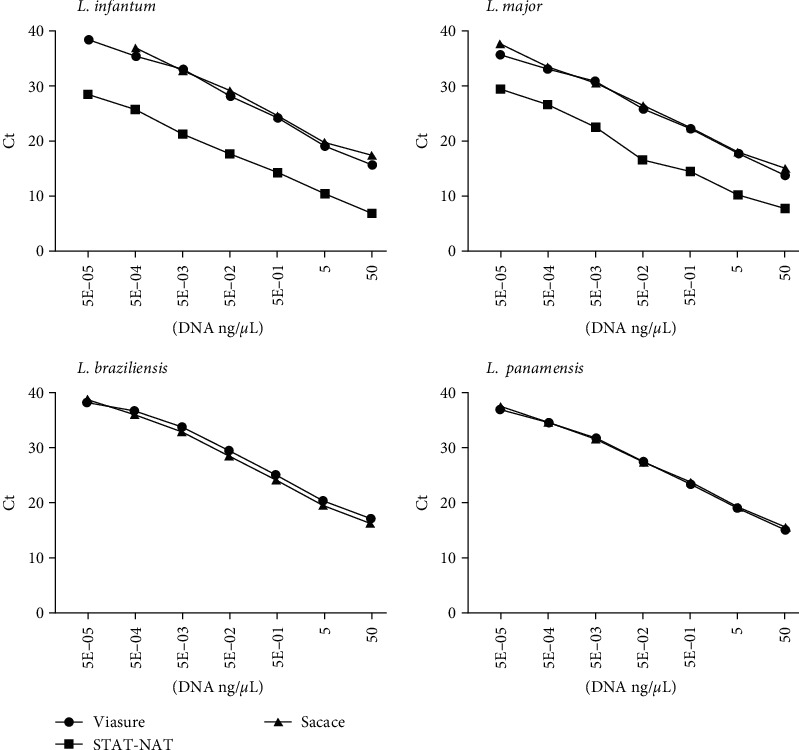
Comparison of the Ct values obtained with the three different kits for *Leishmania infantum*, *L. major*, *L. braziliensis*, and *L. panamensis*. Values correspond to the mean of the duplicates for each dilution.

The Ct values obtained for each assay carried out with different 10-fold dilutions of DNA isolated from the four cultured *Leishmania* species are shown in Figure 1.

The results obtained with the VIASURE prototype could detect all the New and Old World species studied at all the dilution ranges tested in duplicate. Similar results were obtained by the Sacace kit, with the exception of the last dilution point of *L. infantum*, when none of the duplicates were detected. In contrast, the STAT-NAT® kit failed to detect the New World species *L. braziliensis* and *L. panamensis*, although its Ct values for the Old World *Leishmania* species were lower (Figure 1).

### 3.2. Clinical Evaluation

The clinical performance of the STAT-NAT® kit was not assessed, as it failed to detect *Viannia* species. According to the criteria of positivity, 33 out of the 49 samples analyzed were considered positive and 16 negatives (Table 1). The VIASURE prototype achieved a total of 27 qPCR-positive results, whereas the Sacace kit gave positive results in 33 samples. All the positive DNA samples detected by VIASURE were positive by Sacace, but not vice versa. The global results are shown in Table 2, including previous data based on culture methods and the characterization of the species. All the samples with positive cultures tested positive with both assessed kits (VIASURE and Sacace). When using VIASURE, 23 skin biopsies and four samples from VL patients were positive, compared to 28 skin biopsies and five VL samples with Sacace. Both kits showed similar Ct values in positive samples.

The diagnostic accuracy of the VIASURE prototype is shown in Table 3, being the sensitivity of 81.8%.

## 4. Discussion

In Spain, as in other countries of the Mediterranean basin, *L. infantum* is the only autochthonous species that causes VL and CL, including nontypical forms and mucosal involvement [23, 24]. Increased population movements have altered the etiology and distribution of leishmaniasis, which has become increasingly common, including in nonendemic countries [23]. Imported cases of leishmaniasis have been reported in Spain and other Mediterranean countries, especially of CL, involving infection by *Leishmania* species from both the New and Old World [8, 25–28].

According to the WHO, CL incidence in the EU countries affected by leishmaniasis increased significantly in the periods 2005–2008 and 2017–2020 (e.g., from 0.01 to 0.27 in France and from 0.03 to 0.4 in Spain). In contrast, VL has increased in parts of the European Mediterranean region but insignificantly. It should be stressed that CL is not a mandatory reportable disease in many countries, which affects the accuracy of the incidence rate, as many positive diagnoses of *Leishmania* patients are lost. The number of imported cases and the growing incidence in this endemic region could have serious implications for disease management due to an unnoticed spread of nonautochthonous leishmaniasis, increased treatment failure, and development of resistance to treatments [29, 30].

Thus, in the clinical context, there is a pressing need to establish optimized protocols for the detection and identification of all potential etiological agents of leishmaniasis, as this would allow a more accurate prognosis and efficient treatment [23, 31]. In the present work, we assessed the analytical sensitivity of the VIASURE *Leishmania* Real-Time PCR Detection Kit prototype for the detection of the autochthonous *L. infantum* and species from countries of the Old World (*L. major*) and New World (*L. braziliensis* and *L. panamensis*) in comparison with two commercial CE-certified kits: Sacace *Leishmania* spp. Real-TM PCR and Sentinel STAT-NAT® *Leishmania* spp.

The traditional diagnosis of leishmaniasis relies on the microscopic detection of the amastigote form of the parasite in stained samples and *Leishmania* culture, but newer techniques based on PCR assays offer greater sensitivity [32–34]. Of the 49 samples in our study, 13 were positive by culture isolation, whereas 27 and 33 tested positive by the VIASURE and Sacace qPCR kits, respectively. In the case of the STAT-NAT® kit, only DNA samples from CL patients from the Old World and VL cases were positive.

As the causative agents of VL are the species belonging to the *L. donovani* complex [24, 33], its molecular diagnosis is more straightforward compared to TL, which is caused by a wider variety of *Leishmania* species. Designing and standardizing a PCR method for TL diagnosis with sufficient sensitivity and specificity has been a significant challenge due to the requirement of targeting multiple species [20, 21]. Accordingly, we found that the STAT-NAT® kit was sensitive for VL diagnosis, as shown by the Ct obtained, but could not detect CL caused by New World species (see Figure 1).

Overall, PCR-based methods are accessible, safe, have improved/good sensitivity, and provide reliable results in routine laboratory conditions [13]. Another advantage is that parasite culture is not required, and the tests may be applied directly to clinical samples [35]. qPCR assays have been implemented for DNA detection, quantification of parasite burden, and species typing using different targets and protocols. Compared to standard PCR protocols, they are more sensitive and require a simpler standardization procedure [12, 21].

Currently, there is no consensus on a universal protocol for the molecular diagnosis of leishmaniasis, and various molecular targets for *Leishmania* identification have been described for conventional and real-time PCR assays, such as kDNA, ITS-1, SSU, and *hsp70* [20, 36–39]. Although several in-house qPCR assays have been developed, very few studies have focused on validating commercial PCR-based kits for leishmaniasis diagnosis in humans, which are useful for routine laboratory testing. To obtain the necessary certification for marketing, these diagnostic tools need validation by independent studies. In the present study, the STAT-NAT® kit showed better analytical sensitivity than the VIASURE and Sacace kits for the subgenus *Leishmania* (*L. infantum* and *L. major*) but failed to detect species of the New World subgenus *Viannia* (*L. braziliensis* and *L. panamensis*). Consequently, it was not used for clinical evaluation. Similar Ct values were obtained with the VIASURE and Sacace kits, and the detection limit of both tests was comparable, even for analytical sensitivity and specificity. Sacace had a higher diagnostic sensitivity and detected more positive samples (33 versus 27 by the prototype). Nevertheless, as indicated in Section 2, when using the Sacace kit, the extraction control was not added to the clinical samples, as these had been prepared prior to the study, which was retrospective in nature. This could have affected the sensitivity of the assay, as a monoplex reaction is carried out when there is no extraction control. Different studies report reduced sensitivity in multiplex versus monoplex qPCR assays when using samples with a low amount of DNA and high Ct values [40, 41].

The prototype has been able to diagnose both autochthonous and imported cases of CL and VL, which are increasing in the Mediterranean region, caused by different species of Leishmania [23, 25]. It is also worth noting that the patient's epidemiological context and clinical symptoms are very important parameters for the development of treatment once the *Leishmania* infection has been diagnosed. The therapeutic regimen will depend on the clinical presentation, the host's condition, the species causing the infection, and the size of the lesion, among other variables [8–30].

In routine care diagnosis, it is important that results are obtained quickly, using a simple protocol and ready-to-use reagents, and are easy to interpret. Designed to meet these requirements, the VIASURE *Leishmania* Real-Time PCR Kit prototype comes in an 8-strip tube format, in which all the reagents needed for the multiplex qPCR are lyophilized, which allows room temperature transport and storage. After applying a hydration buffer, provided by the manufacturer, only the DNA sample needs to be added to the solution before starting the reaction, avoiding time-consuming manipulation steps, thus reducing the risk of contamination. In contrast, the Sacace *Leishmania* spp. Real-TM PCR Kit comes with different vials for each reagent, and buffer is needed for the qPCR, which clearly lengthens the sample preparation time, and storage must be at −20°C. Interpretation of results was similarly straightforward in both assays.

One of the goals of this study was to assess how the VIASURE kit would behave in routine diagnostic testing for *Leishmania* in different biological samples. This was done using a panel of DNAs from patients with suspected infection by *Leishmania* and different clinical manifestations (CL and VL), as well as DNA extracted from biological samples (45 from skin biopsies, three from bone marrow, one from a liver biopsy, and one from peripheral blood). Both kits were able to detect *Leishmania* in both visceral and cutaneous samples, regardless of the source (autochthonous or imported cases and different human clinical samples).

Notably, one of the limitations of commercialized PCR kits is they do not allow species-specific detection of *Leishmania*. Therefore, future research could focus on developing a single multiplex qPCR assay that is able to differentiate between the most common causal species of human CL.

## 5. Conclusion

The evaluation results of the VIASURE *Leishmania* Real-Time PCR Detection Kit prototype indicate that the test is a promising candidate for routine diagnosis of CL and VL in endemic countries. However, more studies are necessary to address its sensitivity and specificity.

## Figures and Tables

**Table 1:** Final results of the VIASURE *Leishmania* Real-Time PCR Detection Kit according to the interpretation criteria (see Section 2.4).

	Commercial Sacace kit		
	Pos	Neg	Total
VIASURE kit prototype			
Pos	27	0	**27**
Neg	6	16	**22**
Total	**33**	**16**	**49**

**Table 2:** Clinical performance based on analysis of DNA samples (*n* = 49): mean Ct values of the duplicate samples for each qPCR kit.

Sample ID	Culture	VIASURE Ct	SACACE Ct	Clinical form/sample	Species identification ^*∗∗*^	Sample ID	Culture	VIASURE Ct	SACACE Ct	Clinical form/sample	Species identification
**1**	Neg	36.944	35.852	CL/SB		**26** ^*∗*^	Neg	28.818	28.950	CL/SB	
**2**	Neg	Neg	Neg	CL/SB		**27**	Neg	Neg	Neg	CL/SB	
**3**	Neg	Neg	Neg	CL/SB		**28**	Neg	36.274	35.693	CL/SB	
**4**	Neg	Neg	Neg	CL/SB		**29**	Neg	Neg	Neg	CL/SB	
**5**	Neg	Neg	Neg	CL/SB		**30** ^*∗*^	Pos	16.299	16.664	CL/SB	*L. infantum*
**6**	Neg	Neg	Neg	CL/SB		**31**	Neg	32.437	33.767	CL/SB	
**7**	Pos	21.028	19.867	CL/SB	*L. braziliensis*	**32** ^*∗*^	Neg	33.370	34.922	CL/SB	
**8** ^*∗*^	Neg	31.401	31.326	CL/SB		**33**	Neg	38.231	38.497	CL/SB	
**9**	Neg	Neg	Neg	CL/SB		**34**	Pos	33.476	32.940	CL/SB	*L. panamensis*
**10** ^*∗*^	Neg	32.812	33.210	CL/SB		**35**	Neg	Neg	37.799	CL/SB	
**11**	Neg	Neg	37.669	CL/SB		**36**	Neg	Neg	Neg	CL/SB	
**12** ^*∗*^	Pos	20.922	23.568	CL/SB	*L. infantum*	**37** ^*∗*^	Neg	33.867	31.056	CL/SB	
**13** ^*∗*^	Pos	18.029	21.003	CL/SB	*L. infantum*	**38**	Neg	Neg	Neg	CL/SB	
**14** ^*∗*^	Neg	39.649	36.799	CL/SB		**39**	Neg	Neg	Neg	CL/SB	
**15** ^*∗*^	Pos	24.630	25.873	CL/SB	*L. infantum*	**40**	Neg	Neg	Neg	CL/SB	
**16** ^*∗*^	Pos	28.131	28.439	CL/SB	*L. major*	**41** ^*∗*^	Pos	26.841	29.345	CL/SB	*L. infantum*
**17**	Neg	Neg	Neg	CL/SB		**42**	Neg	Neg	Neg	CL/SB	
**18** ^*∗*^	Pos	27.005	27.643	CL/SB	*L. major*	**43**	Neg	Neg	Neg	CL/SB	
**19**	Neg	Neg	36.215	CL/SB		**44**	Neg	Neg	Neg	CL/SB	
**20**	Pos	31.950	30.948	CL/SB	*L. major*	**45** ^*∗*^	Neg	26.485	26.871	VL/LB	
**21**	Neg	Neg	37.315	CL/SB		**46** ^*∗*^	Pos	22.668	22.829	VL/BM	*L. infantum*
**22**	Neg	Neg	37.171	CL/SB		**47** ^*∗*^	Pos	29.201	27.664	VL/BM	*L. infantum*
**23**	Neg	34.787	32.655	CL/SB		**48** ^*∗*^	Pos	32.353	32.874	VL/PB	*L. infantum*
**24**	Neg	29.512	28.445	CL/SB		**49** ^*∗*^	Neg	Neg	36.435	VL/BM	
**25**	Neg	25.604	23.391	CL/SB		**Total POS**	**13/49**	**27/49**	**33/49**		

CL: cutaneous leishmaniasis, VL: visceral leishmaniasis, SB: skin biopsy, BM: bone marrow, LB: liver biopsy, PB: peripheral blood;  ^*∗*^ Positive result by the STAT-NAT® *Leishmania* spp. Kit (Sentinel, Milano, Italy).  ^*∗∗*^ Characterization was done by *hsp70* gene sequencing and MALDI-TOF MS [21].

**Table 3:** Measurements of diagnostic accuracy, expressed in percentage, of the VIASURE *Leishmania* Real-Time PCR Detection Kit prototype.

Measure	Result (%)	95% CI
Sensitivity	81.8	63.92–92.38
Specificity	100	75.93–100
PPV	100	84.50–100
NPV	72.7	49.56–88.39
Prevalence	67.3	52.34–79.64

PPV: positive predictive value; NPV: negative predictive value; CI: confidence interval.

## Data Availability

There are no supporting data in this article.

## References

[1] WHO (2022). *Leishmaniasis—Global Health Observatory: Report of a WHO Consultation of Partners*.

[2] Burza S., Croft S. L., Boelaert M. (2018). Leishmaniasis. *The Lancet*.

[3] Akhoundi M., Kuhls K., Cannet A. (2016). A historical overview of the classification, evolution, and dispersion of *Leishmania* parasites and sandflies. *PLOS Neglected Tropical Diseases*.

[4] de Vries H. J. C., Reedijk S. H., Schallig H. D. F. H. (2015). Cutaneous leishmaniasis: recent developments in diagnosis and management. *American Journal of Clinical Dermatology*.

[5] Bailey M. S., Lockwood D. N. J. (2007). Cutaneous leishmaniasis. *Clinics in Dermatology*.

[6] Alvar J., Vélez I. D., Bern C. (2012). Leishmaniasis worldwide and global estimates of its incidence. *PLOS ONE*.

[7] Steverding D. (2017). The history of leishmaniasis. *Parasites & Vectors*.

[8] Giavedoni P., Iranzo P., Fuertes I., Estrach T., Alsina Gibert M. (2015). Leishmaniasis cutánea. Experiencia de 20 años en un hospital español de tercer nivel. *Actas Dermo-Sifiliográficas*.

[9] Goto H., Lindoso J. A. L. (2014). Current diagnosis and treatment of cutaneous and mucocutaneous leishmaniasis. *Expert Review of Anti-Infective Therapy*.

[10] Mann S., Frasca K., Scherrer S. (2021). A review of leishmaniasis: current knowledge and future directions. *Current Tropical Medicine Reports*.

[11] Aronson N., Herwaldt B. L., Libman M. (2016). Diagnosis and treatment of leishmaniasis: clinical practice guidelines by the Infectious Diseases Society of America (IDSA) and the American Society of Tropical Medicine and Hygiene (ASTMH). *Clinical Infectious Diseases*.

[12] Reimão J. Q., Coser E. M., Lee M. R., Coelho A. C. (2020). Laboratory diagnosis of cutaneous and visceral leishmaniasis: current and future methods. *Microorganisms*.

[13] Thakur S., Joshi J., Kaur S. (2020). Leishmaniasis diagnosis: an update on the use of parasitological, immunological and molecular methods. *Journal of Parasitic Diseases*.

[14] Molina R., Jiménez M., García-Martínez J. (2020). Role of asymptomatic and symptomatic humans as reservoirs of visceral leishmaniasis in a Mediterranean context. *PLOS Neglected Tropical Diseases*.

[15] Chen H., Fan C., Gao H. (2020). Leishmaniasis diagnosis via metagenomic next-generation sequencing. *Frontiers in Cellular and Infection Microbiology*.

[16] Cruz I., Millet A., Carrillo E. (2013). An approach for interlaboratory comparison of conventional and real-time PCR assays for diagnosis of human leishmaniasis. *Experimental Parasitology*.

[17] Galluzzi L., Ceccarelli M., Diotallevi A., Menotta M., Magnani M. (2018). Real-time PCR applications for diagnosis of leishmaniasis. *Parasites & Vectors*.

[18] Ramírez J. C., Cura C. I., da Cruz Moreira O. (2015). Analytical validation of quantitative real-time PCR methods for quantification of *Trypanosoma cruzi* DNA in blood samples from Chagas disease patients. *Journal of Molecular Diagnostics*.

[19] Weirather J. L., Jeronimo S. M. B., Gautam S. (2011). Serial quantitative PCR assay for detection, species discrimination, and quantification of *Leishmania* spp. in human samples. *Journal of Clinical Microbiology*.

[20] de Almeida M. E., Koru O., Steurer F., Herwaldt B. L., da Silva A. J. (2017). Detection and differentiation of *Leishmania* spp. in clinical specimens by use of a SYBR green-based real-time PCR assay. *Journal of Clinical Microbiology*.

[21] Moreira O. C., Yadon Z. E., Cupolillo E. (2018). The applicability of real-time PCR in the diagnostic of cutaneous leishmaniasis and parasite quantification for clinical management: current status and perspectives. *Acta Tropica*.

[22] Herrera G., Barragán N., Luna N. (2020). An interactive database of *Leishmania* species distribution in the Americas. *Scientific Data*.

[23] Fernández-Arévalo A., Ballart C., Muñoz-Basagoiti J. (2022). Autochthonous and imported tegumentary leishmaniasis in Catalonia (Spain): aetiological evolution in the last four decades and usefulness of different typing approaches based on biochemical, molecular and proteomic markers. *Transboundary and Emerging Diseases*.

[24] Pratlong F., Dereure J., Ravel C. (2009). Geographical distribution and epidemiological features of old world cutaneous leishmaniasis foci, based on the isoenzyme analysis of 1048 strains. *Tropical Medicine & International Health*.

[25] Di Muccio T., Scalone A., Bruno A. (2015). Epidemiology of imported leishmaniasis in Italy: implications for a European endemic country. *PLOS ONE*.

[26] Pérez-Ayala A., Norman F., Pérez-Molina J. A., Herrero J. M., Monge B., López-Vélez R. (2009). Imported leishmaniasis: a heterogeneous group of diseases. *Journal of Travel Medicine*.

[27] Söbirk S. K., Inghammar M., Collin M., Davidsson L. (2018). Imported leishmaniasis in Sweden 1993–2016. *Epidemiology and Infection*.

[28] Vandeputte M., van Henten S., van Griensven J. (2020). Epidemiology, clinical pattern and impact of species-specific molecular diagnosis on management of leishmaniasis in Belgium, 2010–2018: a retrospective study. *Travel Medicine and Infectious Disease*.

[29] (2022). Surveillance, prevention and control of leishmaniases in the European Union and its neighbouring countries.

[30] World Health Organization (2010). *Technical Report Series*. 949. *Control of the Leishmaniases*.

[31] Zampieri R. A., Laranjeira-Silva M. F., Muxel S. M., Stocco de Lima A. C., Shaw J. J., Floeter-Winter L. M. (2016). High resolution melting analysis targeting hsp70 as a fast and efficient method for the discrimination of *Leishmania* species. *PLOS Neglected Tropical Diseases*.

[32] Aviles H., Belli A., Armijos R., Monroy F. P., Harris E. (1999). PCR detection and identification of Leishmania parasites in clinical specimens in Ecuador: a comparison with classical diagnostic methods. *The Journal of parasitology*.

[33] de Oliveira C. I., Báfica A., Oliveira F. (2003). Clinical utility of polymerase chain reaction-based detection of *Leishmania* in the diagnosis of American cutaneous leishmaniasis. *Clinical Infectious Diseases*.

[34] Torpiano P., Pace D. (2015). Leishmaniasis: diagnostic issues in Europe. *Expert Review of Anti-Infective Therapy*.

[35] Van der Auwera G., Dujardin J.-C. (2015). Species typing in dermal leishmaniasis. *Clinical Microbiology Reviews*.

[36] de Morais R. C. S., da Costa Oliveira C. N., da Cunha Gonçalves de Albuquerque S. (2016). Real-time PCR for Leishmania species identification: evaluation and comparison with classical techniques. *Experimental Parasitology*.

[37] Gomes C. M., Cesetti M. V., de Paula N. A. (2017). Field validation of SYBR green- and TaqMan-based real-time PCR using biopsy and swab samples to diagnose American Tegumentary Leishmaniasis in an area where *Leishmania* (*Viannia*) *braziliensis* is endemic. *Journal of Clinical Microbiology*.

[38] Hernández C., Alvarez C., González C., Ayala M. S., León C. M., Ramírez J. D. (2014). Identification of six new world *Leishmania* species through the implementation of a high-resolution melting (HRM) genotyping assay. *Parasit and Vectors*.

[39] Prina E., Roux E., Mattei D., Milon G. (2007). *Leishmania* DNA is rapidly degraded following parasite death: an analysis by microscopy and real-time PCR. *Microbes and Infection*.

[40] Thongprachum A., Khamrin P., Pham N. T. K. (2017). Multiplex RT-PCR for rapid detection of viruses commonly causing diarrhea in pediatric patients. *Journal of Medical Virology*.

[41] Veron V., Simon S., Carme B. (2009). Multiplex real-time PCR detection of *P. falciparum*, *P. vivax* and *P. malariae* in human blood samples. *Experimental Parasitology*.

